# Whole-genome sequencing of ocular *Chlamydia trachomatis* isolates from Gadarif State, Sudan

**DOI:** 10.1186/s13071-019-3770-7

**Published:** 2019-11-04

**Authors:** Abdulazeem Abdulsalam Ibrahim Alkhidir, Martin J. Holland, Wafa Ibrahim Elhag, Charlotte A. Williams, Judith Breuer, Abdah Elfatih Elemam, Khalid Mohamed Khalid El Hussain, Mohammed Elfatih Hussein Ournasseir, Harry Pickering

**Affiliations:** 1 Faculty of Medical Laboratory Sciences, Omdurman Islamic University, Omdurman, Sudan; 2 Clinical Research Department, London School of Hygiene & Tropical Medicine, London, UK; 3 Faculty of Medical Laboratory Sciences, Al-Neelain University, Khartoum, Sudan; 4 Division of Infection and Immunity, University College London, London, UK; 5 Microbiology, Virology, and Infection Control, Great Ormond Street Hospital for Children, NHS Foundation Trust, London, UK; 6 Institute of Endemic Diseases, University of Khartoum, Khartoum, Sudan

**Keywords:** Trachoma, Ocular, *Chlamydia trachomatis*, Genomics, Whole-genome sequencing

## Abstract

**Background:**

Trachoma, caused by ocular *Chlamydia trachomatis*, is the leading infectious cause of blindness worldwide. Sudan first reported trachoma in the 1930s and has since been consistently endemic. Ocular *C. trachomatis* previously isolated from trachoma patients in Sudan in 1963 was antigenically identical to an isolate from Saudi Arabia (A/SA1). No contemporary ocular *C. trachomatis* whole genome sequences have been reported from Sudan.

**Methods:**

This study sequenced twenty ocular *C. trachomatis* isolates to improve understanding of pathogen diversity in North-East Africa and examine for genomic variation specific to Sudan, possibly related to the persistence of trachoma in surveyed communities. High quality, whole genome sequences were obtained from 12/20 isolates.

**Results:**

All isolates were serovar A and had *tarP* and *trpA* sequences typical of classical, ocular *C. trachomatis* isolates. The Sudanese isolates formed a closely related subclade within the T2-trachoma clade of *C. trachomatis* phylogeny distinct from geographically disparate ocular isolates, with little intra-population diversity. We found 333 SNPs that were conserved in Sudanese ocular isolates but rare compared to other ocular *C. trachomatis* populations, which were focused in two genomic loci (*CTA0172-CTA0173* and *CTA0482*).

**Conclusions:**

Limited intra-population diversity and geographical clustering of ocular *C. trachomatis* suggests minimal transmission between and slow diversification within trachoma-endemic communities. However, diversity may have been higher pre-treatment in these communities. Over-representation of Sudan-specific SNPs in three genes suggests they may have an impact on *C. trachomatis* growth and transmission in this population.

## Background

*Chlamydia trachomatis* is one of the most common sexually transmitted infections worldwide and the leading infectious cause of blindness. Trachoma, caused by ocular *C. trachomatis* infection, is targeted for elimination by 2020 [1]. Trachoma was first formally described in Sudan in the 1930s [2] and sporadic reports since then [3], including a review of records from 1959 to 1969 [4], indicated trachoma as a public health problem. In Sudan, the causative agent was first isolated from conjunctival scrapings in the 1960s and then again in the 1970s [5, 6], with noted antigenic identity to an historical isolate from Saudi Arabia [7]. In 2011, approximately 100,000 participants were surveyed across the northern states of Sudan [8]. This study identified 14/88 districts requiring antibiotics, facial cleanliness and environmental improvement interventions for trachomatous inflammation, follicular (TF) and 20/88 districts requiring surgery intervention for trachomatous trichiasis (TT). Continued trachoma surveillance and community-level administration of azithromycin have since been undertaken by the Sudanese Ministry of Health as part of the Global Trachoma Mapping Project.

Until recently, few complete genome sequences of ocular *C. trachomatis* have been available [9–11]. Reduced cost and improvements in technique [12–14] have seen a significant increase in whole-genome sequencing (WGS) of *C. trachomatis*; however, most studies have not investigated the relationship between sequence variation and clinical outcomes [15–21]. Studies that have examined this link have invariably focussed on urogenital isolates [22–24]. In 2018, we published a study from Bijagos Islands, Guinea-Bissau that used a genome-wide association study of 81 ocular *C. trachomatis* isolates to identify genomic markers of disease severity in trachoma [25]; this study suggested there is *C. trachomatis* genomic diversity within populations and that it may be linked to clinical outcomes.

Despite the high prevalence of trachoma, no studies have sequenced *C. trachomatis* isolates from Sudan. Trachoma was endemic in the Gadarif districts of Algalabat Eastern (TF: 19.8%; TT: 1.9%) and Alrahad (TF: 7.1%; TT: 4.8%) in 2011. Six and four mass annual rounds of azithromycin, respectively, have had limited impact on trachoma endemicity in these districts, according to the Global Trachoma Atlas (http://www.trachomaatlas.org). A cross-sectional population-based survey was undertaken in these districts to determine the prevalence of active trachoma and ocular *C. trachomatis* infection, as well as the burden of common, nasopharyngeal non-chlamydial pathogens. This study sequenced twenty *C. trachomatis* isolates from the survey in these Sudanese trachoma-endemic districts to characterise ocular *C. trachomatis* genomic diversity.

## Methods

### Study design and population

A descriptive cross-sectional population-based trachoma prevalence study was conducted to determine the prevalence of *C. trachomatis* and active trachoma (TF and/or trachomatous inflammation, intense [TI]) after multiple annual rounds of mass drug administration (MDA) with azithromycin. The studies were undertaken in Jarmai and Gargosha villages of Alrahad District and Alsaraf Alahmar (Bawi East, Bawi West, Bawi South and Bawi Centre) and Saraf Tabaldia villages of Algalabat Eastern District, Gadarif State during the period from November 2016 to April 2019. A total of 3529 children aged 1–9 years were examined for signs of active trachoma.

### Trachoma clinical diagnosis

Examination for trachoma signs was conducted by ophthalmic medical assistants trained in the WHO simplified grading system. Each eye was examined for TF and TI. Both eyes were examined and findings for the worst affected eye recorded. Alcohol was used to clean the examiner’s fingers between examinations. Individuals with signs of active trachoma (TF and/or TI) were offered free treatment with antibiotics according to the national guidelines.

### Sample collection and processing

Four-hundred and nine samples were collected from children clinically diagnosed as having active trachoma (TF and/or TI). Two conjunctival samples were collected from each participant by four passes of a Dacron polyester swab with a one-quarter turn between passes. Swabs were stored in UTM transport media (Thermo Fisher Scientific, Hemel Hempstead, UK) and stored at − 20 °C until processing. Total genomic DNA was extracted from samples using the G-spin Total DNA kit (iNtRON Biotechnology, Seongnam, Korea).

### Detection and quantitation of *C. trachomatis*

A previously validated assay [26, 27] targeting the highly conserved, *C. trachomatis*-specific genomic *omcB* was adapted for use in an end-point PCR to identify *C. trachomatis-*positive samples. Chlamydial DNA from clinical samples was amplified using a conventional PCR machine (SensoQuest, Gränningen, Germany), using Maxime PCR Pre Mix kit (iNtRON Biotechnology, Seongnam, Korea) and primers at 900 nM. Amplification was performed in 30 μl reaction volumes containing 2 μl of template DNA. Cycle conditions were as follows: 95 °C for 30 s, 59.9 °C for 30 s, 72 °C for 2 min. PCR products were subjected to agarose gel electrophoresis. A result was considered positive for *C. trachomatis* when a band of the size 106 bp was visible in the gel. Twenty *C. trachomatis*-positive samples were further tested using an in-house, quantitative ddPCR assay. This assay quantifies both *C. trachomatis* plasmid and genome (*omcB*); *C. trachomatis* load was defined as genome copies per µl.

### Sequencing, processing and analysis of *C. trachomatis*

DNA was enriched using SureSelect *C. trachomatis*-specific baits and sequenced on the Illumina NextSeq platform as previously described [20, 25]. Raw reads were trimmed and filtered using Trimmomatic [28]. Filtered reads were aligned to a reference genome (A/Har13) with Bowtie2 [29], variant calls were identified with SAMtools/BCFtools [30]. Multiple genome and plasmid alignments were generated using progressiveMauve, multiple gene alignments were generated using muscle. Phylogenies were computed using RaxML [31] and visualised in R. Domain structure of *tarP* and truncation of *trpA* were characterised as previously described [25]. Multi-locus sequences types (MLST) were determined from filtered reads using stringMLST [32] and the hr-MLST-6 database [33]. Minimum-spanning trees were constructed using BioNumerics 7.6 created by Applied Maths NV (http://www.applied-maths.com). The discriminatory power of the MLST types was evaluated using Simpson’s discriminatory index as previously described [34]. Pairwise nucleotide diversity was calculated as previously described [25]. ABRicate and the ResFinder database (https://github.com/tseemann/abricate) were used to test for the presence of antimicrobial resistance genes.

### Identification of polymorphisms associated with Sudanese origin

The Sudanese *C. trachomatis* isolates were compared to a global population of ocular isolates (*n* = 166 [15, 17, 20, 21, 25]) to identify polymorphisms associated with Sudanese origin. Sites with a major allele frequency of < 0.8 within the twelve Sudanese isolates and a frequency > 0.2 of the Sudan-conserved alleles within the global population were excluded. Annotations were transferred from the ocular reference genome A/Har13.

## Results

### Demographic information

Twenty *C. trachomatis*-positive samples of sufficient load by ddPCR quantitation of *omcB* load were available for whole-genome sequencing (WGS), from seven villages across two districts of Sudan (Additional file 1: Table S1). All individuals had TF of which 13/20 also had TI. Age and gender were not associated with concurrent TF and TI.

### Sequencing results

Sequencing was successful for all 20 samples (Additional file 1: Table S2), a median of 1.87 × 10^6^ reads were obtained (95% CI: 1.48 × 10^6^–2.50 × 10^6^). A median of 3.73 × 10^5^ reads aligned to the reference genomes, A/HAR-13 (95% CI: 0.09 × 10^5^–17.84 × 10^5^). Based on genome coverage of > 98% and a minimum read depth of 10 there were twelve samples for post-sequencing analysis. *Chlamydia trachomatis* infection load was generally lower in 8/20 samples that did not meet these quality control criteria (mean load 444 *omcB* copies/µl and 1861 *omcB* copies/µl in excluded and included samples respectively). However, two samples from this study with less than 50 *omcB* copies/µl returned high quality sequences, therefore load cannot completely explain sequencing quality. Median read depth of the twelve high quality sequences included in the post-sequencing analysis was 308 (95% CI: 59.9–511.2).

### Phylogenetic analysis

**Fig. 1:**
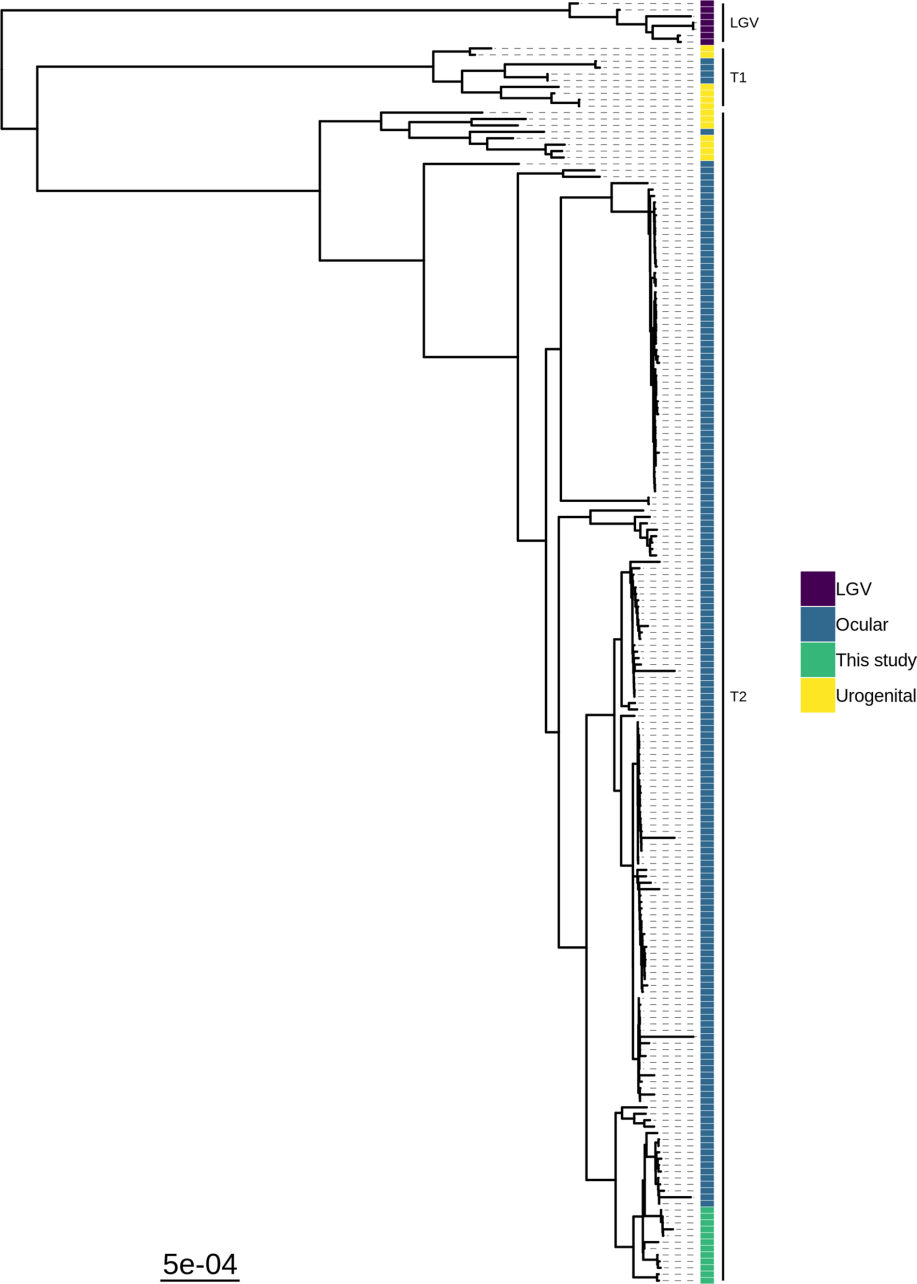
Maximum likelihood reconstruction of whole genome phylogeny of ocular *Chlamydia trachomatis* sequences from Sudan. Whole genome and plasmid phylogeny of 12 *C. trachomatis* sequences from Sudan and 188 *Ct* clinical and reference strains. Sudanese *C. trachomatis* sequences were mapped to *C. trachomatis* A/HAR-13 using Bowtie2. SNPs were called using SAMtools/BCFtools. Phylogenies were computed with RAxML from a variable sites alignment using a GTR + gamma model and are midpoint rooted. The scale-bar indicates evolutionary distance. Sudanese *C. trachomatis* sequences generated in the present study are coloured green, and reference strains are coloured by tissue localization (blue, ocular; yellow, urogenital; purple, LGV)

Phylogenetic analysis of the twelve whole-genome sequences placed them into a closely grouped sub-clade within the T2-trachoma clade (Fig. 1), the closest existing sequences were a sub-clade collected from the Bijagos Islands, Guinea-Bissau in 2012. Plasmid phylogeny showed similar close grouping of the isolates within the trachoma clade (Additional file 2: Figure S1).

**Fig. 2:**
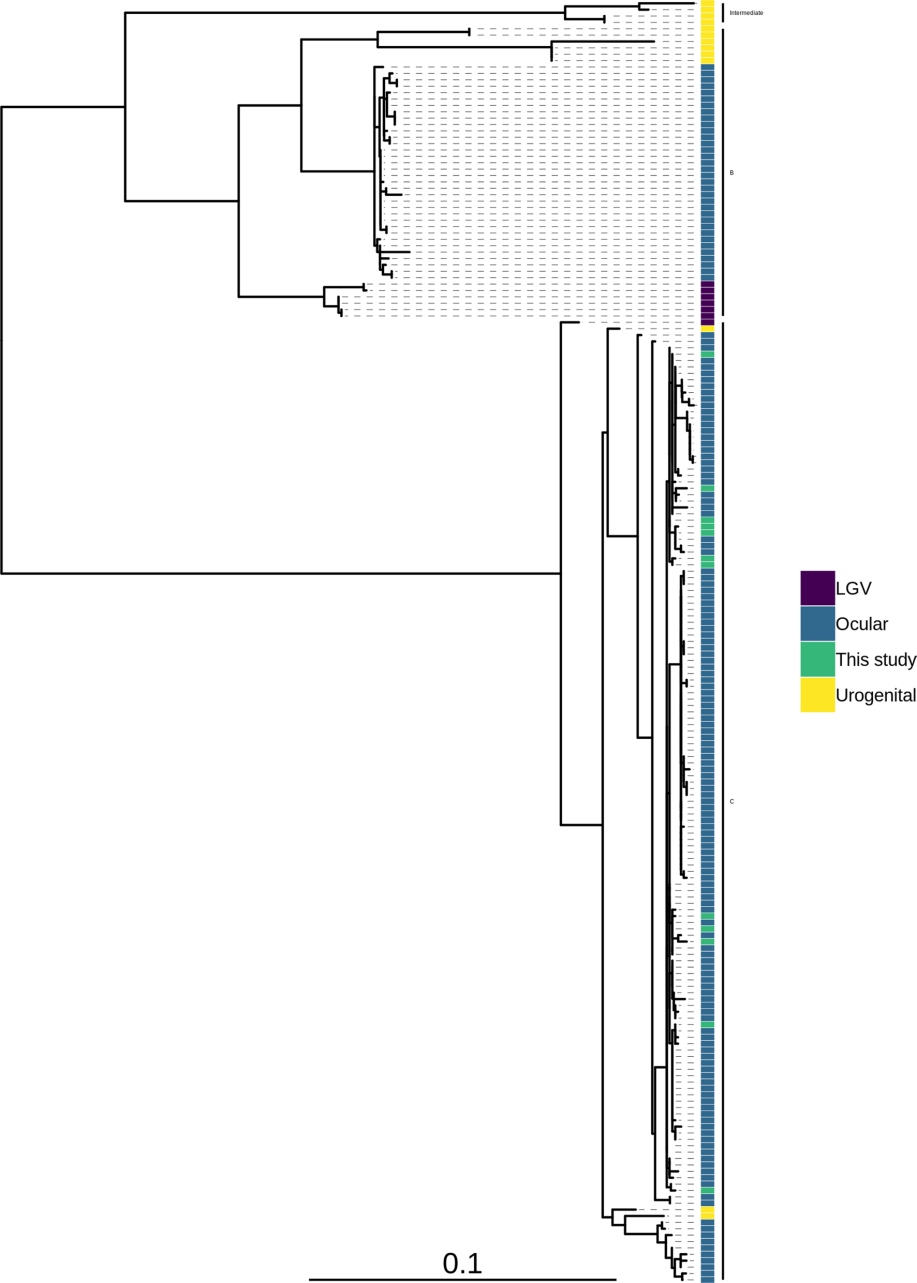
Maximum likelihood reconstruction of *ompA* phylogeny of ocular *Chlamydia trachomatis* sequences from Sudan. Phylogeny of *ompA* from 12 *C. trachomatis* sequences from Sudan and 188 *C. trachomatis* clinical and reference strains. Sudanese *C. trachomatis* sequences were mapped to *C. trachomatis* A/HAR-13 using Bowtie2. SNPs were called using SAMtools/BCFtools. Phylogenies were computed with RAxML from a variable sites alignment using a GTR + gamma model and are midpoint rooted. The scale-bar indicates evolutionary distance. Sudanese *C. trachomatis* sequences generated in the present study are coloured green, and reference strains are coloured by tissue localization (blue, ocular; yellow, urogenital; purple, LGV)

All twelve sequences were *ompA* serovar A (Fig. 2). Seven polymorphic sites were present in *ompA* across nine sequences, leading to four amino acid changes (Table 1). Two sequences contained a single amino acid deletion. The closest related *ompA* sequences by blast+ alignment were A/SA1 (3/12) and A/HAR-13 (9/12).

**Table 1:** Identified *ompA* polymorphisms

Sequences affected	Nucleotide change	Amino acid change	Protein region
B1, J52	305C>T	na	na
A8	349G>A	D117N	Surface exposed
B1, J52	736A>G	na	na
T94, TF16, TF34	743C>T	A248V	Surface exposed
J52	1000T>G	S334A	Membranous
B13	1007A>G	T337A	Surface exposed
A8, TF54	1007_1009del	na	na
TF34	1011A>C	na	na

*Abbreviation*: na, the polymorphism did not lead to an amino acid change and therefore did not affect any region of the protein

**Fig. 3:**
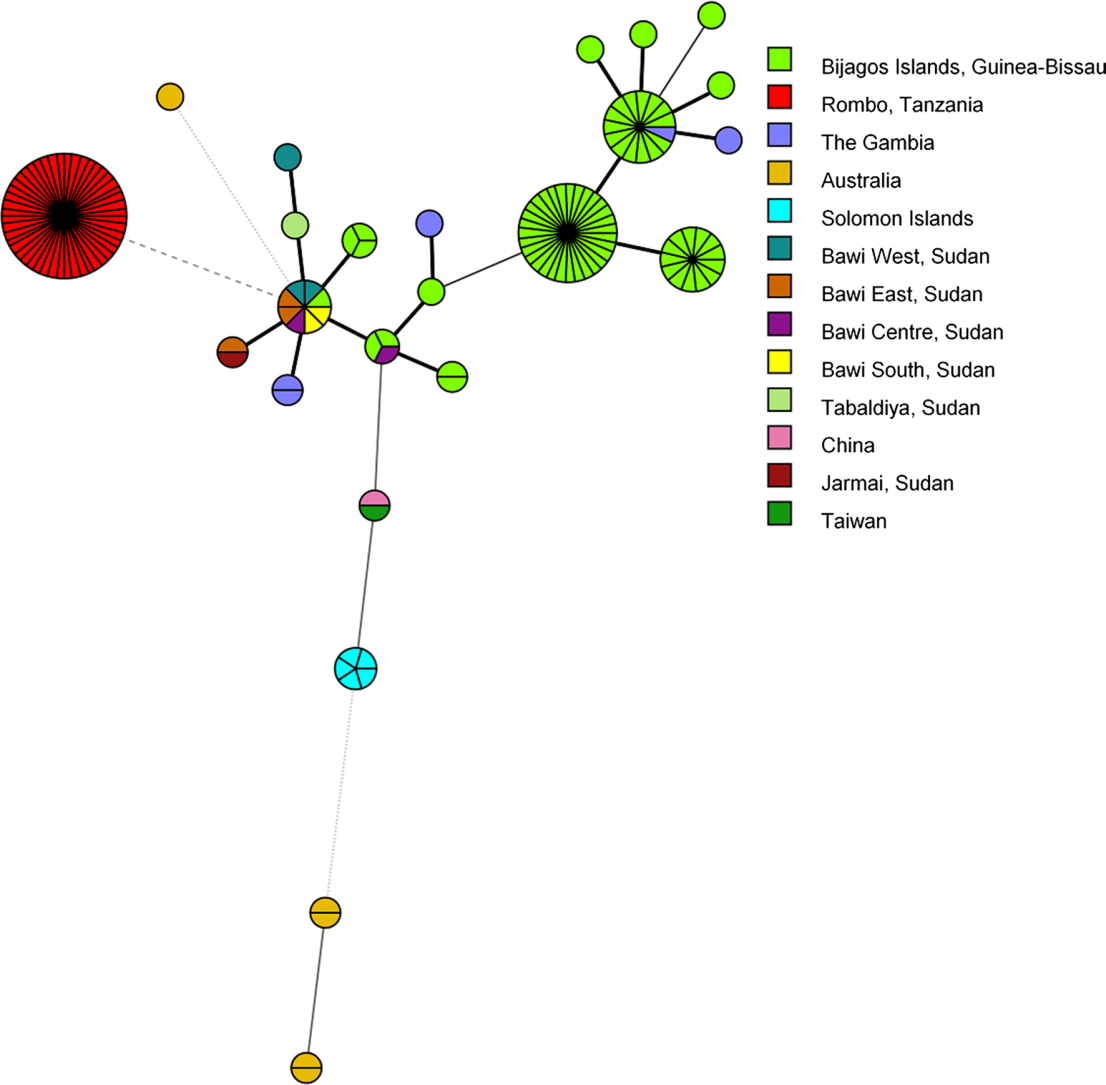
Minimum spanning tree of hr-MLST-6 types of *Chlamydia trachomatis* sequences from Sudan. Twelve *C. trachomatis* sequences from Sudan and 136 ocular *C. trachomatis* clinical and reference strains were used to construct a minimum spanning tree of hr-MLST-6 types. Multi-locus sequence types were determined using stringMLST. Minimum spanning trees were constructed using BioNumerics 7.6. Sudanese sequence types are coloured by village of origin, clinical and reference strains are coloured by country of origin

MLST analysis, including *ompA* (hr-MLST-6), identified four novel sequence types (ST) with a Simpson’s discriminatory index of 0.67. A minimum spanning tree including all available ocular ST showed clustering of Sudanese isolates, with little evidence for village-level resolution (Fig. 3). Pairwise nucleotide diversity using WGS data was 0.0014. All sequences had *tarP* domain structure (four actin-binding domains and three tyrosine-repeat regions) and truncated *trpA* (531del) typical of ocular strains. One sequence had an insertion in *trpA* (115_116AG in B9) which led to an earlier truncation. There was no evidence for the presence of macrolide resistance alleles.

**Fig. 4:**
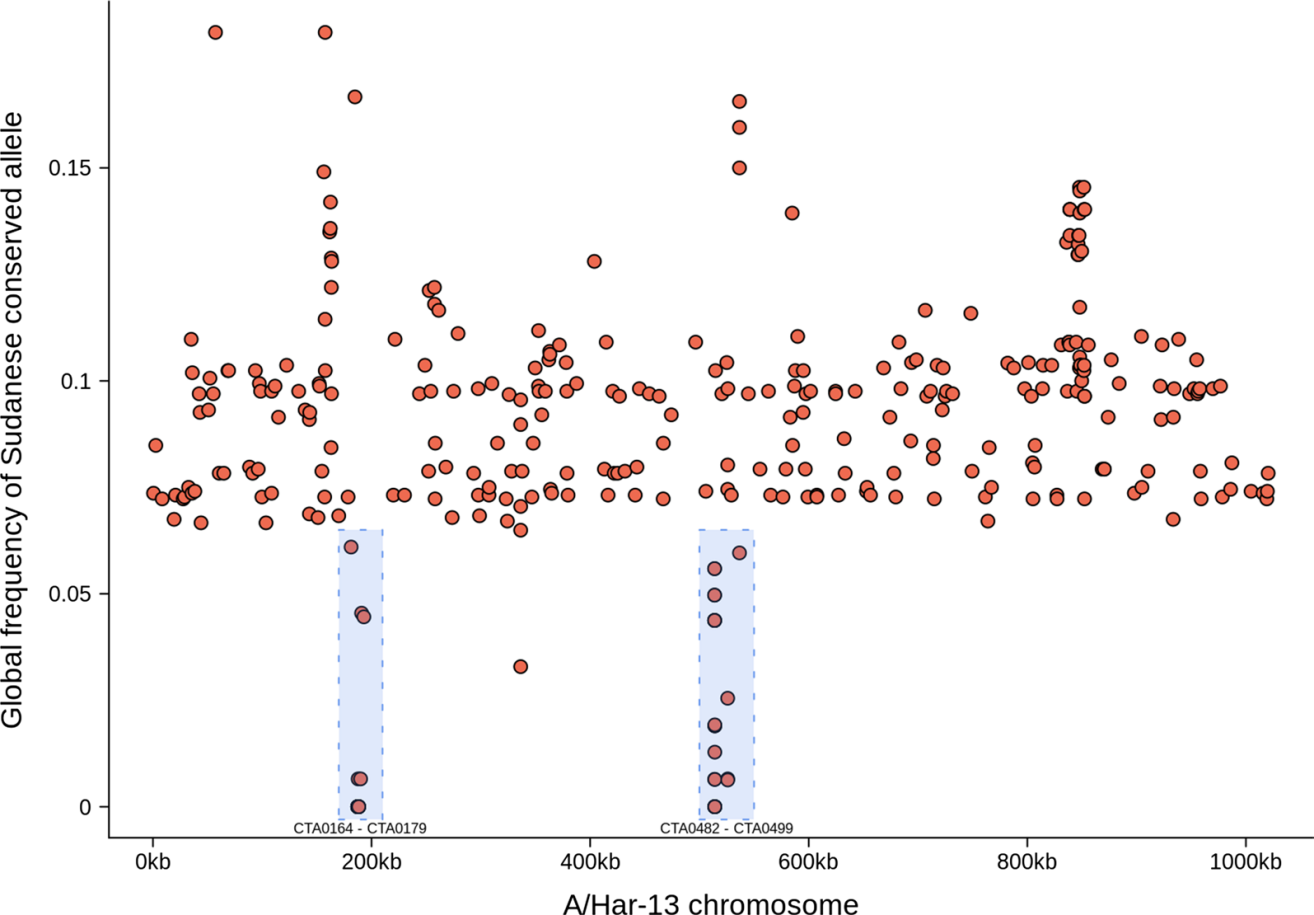
Single nucleotide polymorphisms on the *Chlamydia trachomatis* genome specific to Sudan (*n* = 333). Single nucleotide polymorphisms conserved in Sudan (allele frequency ≥ 0.8) and rare in other *C. trachomatis* isolates (allele frequency ≤ 0.2) were identified by comparing these *C. trachomatis* sequences (*n* = 12) to ocular isolates from other populations (*n* = 166). Two loci (*CTA0172-CTA0173* and *CTA0482*) which harboured the majority of Sudan-specific alleles are indicated (blue boxes)

A comparison of the Sudanese sequences with 166 previously sequenced samples from trachoma-endemic communities [15, 17, 20, 21, 25] identified genomic markers specific to Sudan. After filtering, 333 single nucleotide polymorphisms (SNPs) across 178 sequences were found to be conserved in Sudan (allele frequency ≥ 0.8) and rare in the global population (allele frequency ≤ 0.2). SNPs were dispersed throughout the genome, with two foci in the genes *CTA0164-CTA0179* and *CTA482-CTA499* (Fig. 4). Within these focal regions, *CTA0482* (D/UW3; *CT442*) contained 19 SNPs, *CTA0172* and *CTA0173* (D/UW3; both *CT163*) contained 20 SNPs. A further cluster of SNPs was located between *CTA_0777* and *CTA_0801*, the SNPs in this region were not overrepresented in any individual gene.

## Discussion

This study successfully sequenced twelve recent ocular *C. trachomatis* samples from a trachoma-endemic region of Sudan with no prior characterisation of chlamydial genomics. All sequences were phylogenetically within the T2-trachoma clade and contained *ompA*, *tarP* and *trpA* sequences typical of classical ocular strains. The Sudanese sequences were phylogenetically distinct from trachoma sequences collected in geographically disparate sites. This study found 333 alleles conserved within Sudan and rare within the global ocular *C. trachomatis* population were focussed in two distinct genomic regions. There was no evidence of macrolide resistance alleles in the *C. trachomatis* population.

All sequences were genovar A by *ompA* typing with a high level of conservation, historically this has been the most prevalent ocular *ompA* type in sub-Saharan Africa [15, 25, 35–37]. Whilst three quarters of non-synonymous SNPs in *ompA* were within surface-exposed domains, none were within reported antigenic sites [38–44]. Sequence variation in *tarP* and the tryptophan operon are also ocular clade-specific. There were ten unique *tarP* sequences in this population, all coded for the domain structure typical of ocular isolates, specifically four actin-binding domains and three tyrosine-repeat regions [45]. The sequence of *trpA* was highly conserved, 11 out of 12 identical sequences had a truncating deletion and one had a truncating insertion. Therefore, all of the Sudanese sequences had a non-functional tryptophan operon, thought to be restrictive to growth in the urogenital tract [46, 47]. These features and branching of the Sudanese sequences within the classical T2-trachoma clade suggests they are typical ocular strains. Results of the comparison to a global population of *C. trachomatis* sequences, aimed at identifying Sudan-specific polymorphisms, supported this assertion. Only 333 alleles conserved within Sudan and rare within the global population were found, of which only two were unique to the Sudanese sequences. Two genes, *CTA0172-CTA0173* and *CTA0482*, harboured > 10% of these alleles. Both encoded proteins have been associated with lipid droplets in *C. trachomatis*-infected cells *in vitro*, targeting of which is thought to enhance *C. trachomatis* survival and replication [48, 49]. It is possible that altered expression or activity of these genes may impact the growth and survival of these Sudanese ocular strains.

Pairwise nucleotide diversity is a measure of the extent of polymorphism within a population, a higher value indicating increased polymorphism. Pairwise diversity reported from studies of ocular *C. trachomatis* from different trachoma-endemic communities has produced contrasting results, with those sequences originating directly from ocular swabs being significantly more variable at the population level than those derived from repeatedly passaged cultured isolates. The pairwise diversity in this population was 0.0014, which is higher than isolates from Rombo, Tanzania [50] but lower than found in the Bijagos Islands, Guinea-Bissau [51]. This supports our previous assertion that *in vitro* passage of isolates prior to sequencing, influences sequence diversity. This suggests that in the future, when possible, *C. trachomatis* samples should be sequenced directly from clinical samples.

MLST analysis has been evaluated extensively in urogenital *C. trachomatis*, with evidence suggesting it may be a useful tool for determining diversity in a population [52]. Only one study has investigated its utility in ocular *C. trachomatis* and it focussed on a small number of strains [53], primarily historical reference isolates. Our study identified five novel sequence types. Simpson’s discriminatory index, which calculates the probability of two randomly sampled strains in a population being the same ST, has been used to evaluate the discriminatory power of MLST schemes. The five novel ST identified in this study had a discriminatory index of 0.67, considerably below the suggested threshold of 0.90 for high confidence that the typing system is of sufficient resolution [34]. This was supported by close clustering and overlap of ST between villages from separate districts. The discriminatory index for Sudanese samples is slightly less than that calculated from a global population of trachoma isolates (0.772) and considerably lower than that for a global population of urogenital isolates (0.968) [53]. This is unsurprising as the metric was designed for “large and representative (nonlocal) collections of distinct strains” [34]. The MLST scheme applied in this analysis, which targets five non-housekeeping genes and approximately half of the sequence of *ompA*, provided lower resolution in this case than full-length *ompA* alone (discriminatory index of 0.773). High levels of recombination in and around *ompA* has led others to suggest it is an unsuitable target for molecular epidemiological characterisation of *C. trachomatis* isolates [17], supporting the greater use of WGS and need for exploration of novel MLST systems.

Considering the lack of diversity within the Sudanese sequences, the clear phylogenetic separation from geographically disparate populations of ocular *C. trachomatis* whole genome sequences is striking. This mirrors previous findings from Guinea-Bissau [25], Tanzania [17] and the Solomon Islands [20], suggesting this geographical clustering of sequences is a common feature of ocular *C. trachomatis*. The sequences from Guinea-Bissau (beginning with 11151, 13108 or 9471) are the largest published collection of ocular *C. trachomatis* yet still split into only two sub-clades, one of which branched phylogenetically close to the *C. trachomatis* sequenced in this study. The close relatedness of the Sudanese sequences, collected in 2018, to an isolate collected in Saudi Arabia in 1957 (A/SA1) is even more remarkable. A similar phylogenetic relatedness was found for two isolates collected in The Gambia over 20 years apart (B/Jali-20 and B-M48). These findings suggest slow and geography-related diversification of ocular *C. trachomatis*, with little evidence of transmission between geographically separate trachoma-endemic communities. This may be because *C. trachomatis* is a successful, well-adapted pathogen requiring little further adaptation to maintain within a population or that country/region-specific pressures may be driving adaptation. It is also possible that diversity of *C. trachomatis* in these regions of Sudan had been reduced by prior mass community-level treatment. Although, despite repeated rounds of treatment and consistent with previous studies, no evidence of macrolide resistance was found in this population. This supports results from *ompA* typing of *C. trachomatis* samples pre- and post-treatment that found no difference in diversity [54].

Thus far no study has published whole-genome sequence data from ocular *C. trachomatis* samples collected pre- and post-treatment in the same community. However, studies of ocular *C. trachomatis* sequences have found no change in azithromycin susceptibility after treatment [55–57]. This supports the absence of macrolide resistance genes in our sequences from Sudan. Azithromycin is known to effectively clear infections at the individual level, but ocular *C. trachomatis* often persists in communities even after multiple rounds of treatment [58, 59]. This is likely due to a combination of factors, including baseline levels of infection, environmental improvements and treatment coverage. It is possible that genomic factors may support continued transmission of *C. trachomatis* after treatment, even in the absence of genes that directly inhibit macrolide activity. Genes with critical functions that promote *C. trachomatis* survival and replication may lead to a higher pre-treatment load of infection, reducing the likelihood of complete clearance, or enhance emergence of post-treatment residual infections. Additionally, there is the possibility of indirect resistance in which a resistant population of bacteria can provide protection for a susceptible population [60].

## Conclusions

This first WGS study of ocular *C. trachomatis* from trachoma-endemic regions of Sudan identified typical T2-trachoma isolates with low intra-population diversity and remarkable similarity to a reference *C. trachomatis* strain collected in Saudi Arabia 60 years previously. There was no evidence of macrolide resistance alleles in our *C. trachomatis* sequences from post-treatment communities, however, two foci of polymorphism specific to these populations were identified. A greater sample size and pre-treatment samples are required to reliably investigate if genomic diversity is related to population treatment success. The phylogenetic clustering of sequences by country of collection warrants further investigation to understand the evolutionary history of ocular *C. trachomatis*.

## Supplementary information

**Additional file 1: Table S1.** Patient demographics. **Table S2.** *Chlamydia trachomatis* detection and sequencing.

**Additional file 2: Figure S1.** Maximum likelihood reconstruction of plasmid phylogeny of ocular *Chlamydia trachomatis* sequences from Sudan. Plasmid phylogeny of 12 *C. trachomatis* sequences from Sudan and 188 Ct clinical and reference strains. Sudanese *C. trachomatis* sequences were mapped to *C. trachomatis* A/HAR-13 using Bowtie2. SNPs were called using SAMtools/BCFtools. Phylogenies were computed with RAxML from a variable sites alignment using a GTR + gamma model and are midpoint rooted. The scale-bar indicates evolutionary distance. Sudanese *C. trachomatis* sequences in this study are coloured green, and reference strains are coloured by tissue localization (blue, ocular; yellow, urogenital; purple, LGV).

## Data Availability

All sequence data are available from the European Bioinformatics Institute (EBI) short read archive (PRJEB32246).

## References

[1] World Health Assembly (1998). Global Elimination of Blinding Trachoma. 51st World Health Assembly, Geneva, 16 May 1998, Resolution WHA51,.

[2] Maccallan AF (1934). Trachoma in the British colonial empire. Its relation to blindness; the existing means of relief; means of prophylaxis. Br J Ophthalmol..

[3] Majcuk JF (1966). A study of trachoma and associated infections in the Sudan. Bull World Health Organ..

[4] Salim AR, Sheikh HA (1975). Trachoma in the Sudan. An epidemiological study. Br J Ophthalmol..

[5] Harper IA (1963). Isolation of trachoma virus in the Sudan. Lancet..

[6] Salim AR, Sheikh HA (1975). Trachoma in the Sudan. A laboratory study. Br J Ophthalmol..

[7] Murray ES, Bell SD, Hanna AT, Nichols RL, Snyder JC (1960). Studies on trachoma. 1. Isolation and identification of strains of elementary bodies from Saudi Arabia and Egypt. Am J Trop Med Hyg..

[8] Hassan A, Ngondi JM, King JD, Elshafie BE, Al Ginaid G, Elsanousi M (2011). The prevalence of blinding trachoma in northern states of Sudan. PLoS Negl Trop Dis..

[9] Stephens RS, Kalman S, Lammel C, Fan J, Marathe R, Aravind L (1998). Genome sequence of an obligate intracellular pathogen of humans: *Chlamydia trachomatis*. Science..

[10] Carlson JH, Porcella SF, McClarty G, Caldwell HD (2005). Comparative genomic analysis of *Chlamydia trachomatis* oculotropic and genitotropic strains. Infect Immun..

[11] Thomson NR, Holden MT, Carder C, Lennard N, Lockey SJ, Marsh P (2008). *Chlamydia trachomatis*: genome sequence analysis of lymphogranuloma venereum isolates. Genome Res..

[12] Seth-Smith HM, Harris SR, Skilton RJ, Radebe FM, Golparian D, Shipitsyna E (2013). Whole-genome sequences of *Chlamydia trachomatis* directly from clinical samples without culture. Genome Res..

[13] Christiansen MT, Brown AC, Kundu S, Tutill HJ, Williams R, Brown JR (2014). Whole-genome enrichment and sequencing of *Chlamydia trachomatis* directly from clinical samples. BMC Infect Dis..

[14] Brown AC, Christiansen MT (2017). Whole-genome enrichment using RNA probes and sequencing of *Chlamydia trachomatis* directly from clinical samples. Methods Mol Biol..

[15] Harris SR, Clarke IN, Seth-Smith HM, Solomon AW, Cutcliffe LT, Marsh P (2012). Whole-genome analysis of diverse *Chlamydia trachomatis* strains identifies phylogenetic relationships masked by current clinical typing. Nat Genet..

[16] O’Neill CE, Seth-Smith HM, Van Der Pol B, Harris SR, Thomson NR, Cutcliffe LT (2013). *Chlamydia trachomatis* clinical isolates identified as tetracycline resistant do not exhibit resistance *in vitro*: whole-genome sequencing reveals a mutation in porB but no evidence for tetracycline resistance genes. Microbiology..

[17] Hadfield J, Harris SR, Seth-Smith HMB, Parmar S, Andersson P, Giffard PM (2017). Comprehensive global genome dynamics of *Chlamydia trachomatis* show ancient diversification followed by contemporary mixing and recent lineage expansion. Genome Res..

[18] Peters RPH, Doyle R, Redelinghuys MJ, McIntyre JA, Verjans GM, Breuer J (2017). *Chlamydia trachomatis* Biovar L2 infection in women in South Africa. Emerg Infect Dis..

[19] Eder T, Kobus S, Stallmann S, Stepanow S, Kohrer K, Hegemann JH (2017). Genome sequencing of *Chlamydia trachomatis* serovars E and F reveals substantial genetic variation. Pathog Dis..

[20] Butcher RM, Sokana O, Jack K, Macleod CK, Marks ME, Kalae E (2016). Low prevalence of conjunctival infection with *Chlamydia trachomatis* in a treatment-naive trachoma-endemic region of the Solomon Islands. PLoS Negl Trop Dis..

[21] Andersson P, Harris SR, Seth Smith HM, Hadfield J, O’Neill C, Cutcliffe LT (2016). *Chlamydia trachomatis* from Australian Aboriginal people with trachoma are polyphyletic composed of multiple distinctive lineages. Nat Commun..

[22] Jeffrey BM, Suchland RJ, Quinn KL, Davidson JR, Stamm WE, Rockey DD (2010). Genome sequencing of recent clinical *Chlamydia trachomatis* strains identifies loci associated with tissue tropism and regions of apparent recombination. Infect Immun..

[23] Suchland RJ, Dimond ZE, Putman TE, Rockey DD (2017). Demonstration of persistent infections and genome stability by whole-genome sequencing of repeat-positive, same-serovar *Chlamydia trachomatis* collected from the female genital tract. J Infect Dis..

[24] Versteeg B, Bruisten SM, Pannekoek Y, Jolley KA, Maiden MCJ, van der Ende A (2018). Genomic analyses of the *Chlamydia trachomatis* core genome show an association between chromosomal genome, plasmid type and disease. BMC Genomics..

[25] Last AR, Pickering H, Roberts CH, Coll F, Phelan J, Burr SE (2018). Population-based analysis of ocular *Chlamydia trachomatis* in trachoma-endemic West African communities identifies genomic markers of disease severity. Genome Med..

[26] Roberts CH, Last A, Molina-Gonzalez S, Cassama E, Butcher R, Nabicassa M (2013). Development and evaluation of a next-generation digital PCR diagnostic assay for ocular *Chlamydia trachomatis* infections. J Clin Microbiol..

[27] Butcher R, Houghton J, Derrick T, Ramadhani A, Herrera B, Last AR (2017). Reduced-cost *Chlamydia trachomatis*-specific multiplex real-time PCR diagnostic assay evaluated for ocular swabs and use by trachoma research programmes. J Microbiol Methods..

[28] Bolger AM, Lohse M, Usadel B (2014). Trimmomatic: a flexible trimmer for Illumina sequence data. Bioinformatics..

[29] Langmead B, Salzberg SL (2012). Fast gapped-read alignment with Bowtie 2. Nat Methods..

[30] Li H, Handsaker B, Wysoker A, Fennell T, Ruan J, Homer N (2009). The sequence alignment/map format and SAMtools. Bioinformatics..

[31] Stamatakis A (2014). RAxML version 8: a tool for phylogenetic analysis and post-analysis of large phylogenies. Bioinformatics..

[32] Gupta A, Jordan IK, Rishishwar L (2017). stringMLST: a fast k-mer based tool for multilocus sequence typing. Bioinformatics..

[33] Versteeg B, Bruisten SM, van der Ende A, Pannekoek Y (2016). Does typing of *Chlamydia trachomatis* using housekeeping multilocus sequence typing reveal different sexual networks among heterosexuals and men who have sex with men?. BMC Infect Dis..

[34] Hunter PR, Gaston MA (1988). Numerical index of the discriminatory ability of typing systems: an application of Simpsonʼs index of diversity. J Clin Microbiol..

[35] Hayes LJ, Bailey RL, Mabey DC, Clarke IN, Pickett MA, Watt PJ (1992). Genotyping of *Chlamydia trachomatis* from a trachoma-endemic village in the Gambia by a nested polymerase chain reaction: identification of strain variants. J Infect Dis..

[36] Hsieh YH, Bobo LD, Quinn TC, West SK (2001). Determinants of trachoma endemicity using *Chlamydia trachomatis* ompA DNA sequencing. Microbes Infect..

[37] Andreasen AA, Burton MJ, Holland MJ, Polley S, Faal N, Mabey DC (2008). *Chlamydia trachomatis* ompA variants in trachoma: what do they tell us?. PLoS Negl Trop Dis..

[38] Conlan JW, Ferris S, Clarke IN, Ward ME (1989). Surface-exposed epitopes on the major outer-membrane protein of *Chlamydia trachomatis* defined with peptide antisera. J Gen Microbiol..

[39] Su H, Morrison RP, Watkins NG, Caldwell HD (1990). Identification and characterization of T helper cell epitopes of the major outer membrane protein of *Chlamydia trachomatis*. J Exp Med..

[40] Ortiz L, Demick KP, Petersen JW, Polka M, Rudersdorf RA, Van der Pol B (1996). *Chlamydia trachomatis* major outer membrane protein (MOMP) epitopes that activate HLA class II-restricted T cells from infected humans. J Immunol..

[41] Arno JN, Xie C, Jones RB, Van Der Pol B (1998). Identification of T cells that respond to serovar-specific regions of the *Chlamydia trachomatis* major outer membrane protein in persons with serovar E infection. J Infect Dis..

[42] Zhu S, Chen J, Zheng M, Gong W, Xue X, Li W (2010). Identification of immunodominant linear B-cell epitopes within the major outer membrane protein of *Chlamydia trachomatis*. Acta Biochim Biophys Sin (Shanghai)..

[43] Tifrea DF, Pal S, Popot JL, Cocco MJ, de la Maza LM (2014). Increased immunoaccessibility of MOMP epitopes in a vaccine formulated with amphipols may account for the very robust protection elicited against a vaginal challenge with *Chlamydia muridarum*. J Immunol..

[44] Jiang P, Cai Y, Chen J, Ye X, Mao S, Zhu S (2017). Evaluation of tandem *Chlamydia trachomatis* MOMP multi-epitopes vaccine in BALB/c mice model. Vaccine..

[45] Lutter EI, Bonner C, Holland MJ, Suchland RJ, Stamm WE, Jewett TJ (2010). Phylogenetic analysis of *Chlamydia trachomatis* Tarp and correlation with clinical phenotype. Infect Immun..

[46] Caldwell HD, Wood H, Crane D, Bailey R, Jones RB, Mabey D (2003). Polymorphisms in *Chlamydia trachomatis* tryptophan synthase genes differentiate between genital and ocular isolates. J Clin Invest..

[47] OʼNeill CE, Skilton RJ, Pearson SA, Filardo S, Andersson P, Clarke IN (2018). Genetic transformation of a *C. trachomatis* ocular isolate with the functional tryptophan synthase operon confers an indole-rescuable phenotype. Front Cell Infect Microbiol.

[48] Kumar Y, Cocchiaro J, Valdivia RH (2006). The obligate intracellular pathogen *Chlamydia trachomatis* targets host lipid droplets. Curr Biol..

[49] Saka HA, Thompson JW, Chen YS, Dubois LG, Haas JT, Moseley A (2015). *Chlamydia trachomatis* infection leads to defined alterations to the lipid droplet proteome in epithelial cells. PLoS One..

[50] Solomon AW, Holland MJ, Burton MJ, West SK, Alexander ND, Aguirre A (2003). Strategies for control of trachoma: observational study with quantitative PCR. Lancet..

[51] Last AR, Burr SE, Weiss HA, Harding-Esch EM, Cassama E, Nabicassa M (2014). Risk factors for active trachoma and ocular *Chlamydia trachomatis* infection in treatment-naive trachoma-hyperendemic communities of the Bijagos Archipelago, Guinea Bissau. PLoS Negl Trop Dis..

[52] Patino LH, Camargo M, Munoz M, Rios-Chaparro DI, Patarroyo MA, Ramirez JD (2018). Unveiling the multilocus sequence typing (MLST) schemes and core genome phylogenies for genotyping *Chlamydia trachomatis*. Front Microbiol..

[53] Herrmann B, Isaksson J, Ryberg M, Tangrot J, Saleh I, Versteeg B (2015). Global multilocus sequence type analysis of *Chlamydia trachomatis* strains from 16 countries. J Clin Microbiol..

[54] Chin SA, Morberg DP, Alemayehu W, Melese M, Lakew T, Chen MC (2018). Diversity of *Chlamydia trachomatis* in trachoma-hyperendemic communities treated with azithromycin. Am J Epidemiol..

[55] Solomon AW, Mohammed Z, Massae PA, Shao JF, Foster A, Mabey DC (2005). Impact of mass distribution of azithromycin on the antibiotic susceptibilities of ocular *Chlamydia trachomatis*. Antimicrob Agents Chemother..

[56] Hong KC, Schachter J, Moncada J, Zhou Z, House J, Lietman TM (2009). Lack of macrolide resistance in *Chlamydia trachomatis* after mass azithromycin distributions for trachoma. Emerg Infect Dis..

[57] West SK, Moncada J, Munoz B, Mkocha H, Storey P, Hardick J (2014). Is there evidence for resistance of ocular *Chlamydia trachomatis* to azithromycin after mass treatment for trachoma control?. J Infect Dis..

[58] West ES, Munoz B, Mkocha H, Holland MJ, Aguirre A, Solomon AW (2005). Mass treatment and the effect on the load of *Chlamydia trachomatis* infection in a trachoma-hyperendemic community. Invest Ophthalmol Vis Sci..

[59] Nash SD, Stewart AEP, Zerihun M, Sata E, Gessese D, Melak B (2018). Ocular *Chlamydia trachomatis* infection under the surgery, antibiotics, facial cleanliness, and environmental improvement strategy in Amhara, Ethiopia, 2011–2015. Clin Infect Dis..

[60] Nicoloff H, Andersson DI (2016). Indirect resistance to several classes of antibiotics in cocultures with resistant bacteria expressing antibiotic-modifying or -degrading enzymes. J Antimicrob Chemother..

