# Supplementary material for: Whole-genome sequencing of ocular Chlamydia trachomatis isolates from Gadarif State, Sudan
Source: Parasit Vectors. 2019 Nov 4;12:518. doi: 10.1186/s13071-019-3770-7 (PMC6829945; doi:10.1186/s13071-019-3770-7)
Supplement: Supplementary file 1 — Additional file 1: Table S1. Patient demographics. Table S2. Chlamydia trachomatis detection and sequencing. [file 13071_2019_3770_MOESM1_ESM.docx]

**Whole-genome sequencing of ocular *Chlamydia trachomatis* isolates from Gadarif state, Sudan**

**Additional file 1: Table S1.** Patient demographics

| **Isolate** | **Age (years)** | **Gender** | **Village** | **Locality** | **Trachoma phenotype** |
| --- | --- | --- | --- | --- | --- |
| B1 | 9 | Male | Bawi East | Algalabat Eastern | TF/TI |
| B9 | 4 | Female | Bawi East | Algalabat Eastern | TF/TI |
| B13 | 7 | Female | Bawi East | Algalabat Eastern | TF/TI |
| S35 | 4 | Male | Bawi Centre | Algalabat Eastern | TF |
| S45 | 3 | Male | Bawi Centre | Algalabat Eastern | TF |
| S59 | 1 | Female | Bawi Centre | Algalabat Eastern | TF/TI |
| TF16 | 4 | Female | Bawi West | Algalabat Eastern | TF |
| TF34 | 6 | Female | Bawi West | Algalabat Eastern | TF |
| TF54 | 2 | Male | Bawi West | Algalabat Eastern | TF/TI |
| G10 | 4 | Male | Gargosha | Alrahad | TF/TI |
| G19 | 4 | Male | Gargosha | Alrahad | TF/TI |
| G24 | 5 | Female | Gargosha | Alrahad | TF/TI |
| T8 | 7 | Female | Tabaldia | Algalabat Eastern | TF |
| T64 | 4 | Male | Tabaldia | Algalabat Eastern | TF/TI |
| T94 | 6 | Female | Tabaldia | Algalabat Eastern | TF/TI |
| J47 | 9 | Female | Jarmai | Alrahad | TF |
| J52 | 4 | Male | Jarmai | Alrahad | TF/TI |
| A8 | 7 | Male | Bawi South | Algalabat Eastern | TF/TI |
| A9 | 6 | Female | Bawi South | Algalabat Eastern | TF |
| A15 | 3 | Female | Bawi South | Algalabat Eastern | TF/TI |

**Additional file 1: Table S2.** Ct detection and sequencing

| **Isolate** | **Ct load***  **(omcB copies per ul)** | **Read count** | **Reads aligning to reference** (%)** | **Genome coverage*** (%)** | **Median read depth (95% CI)** |
| --- | --- | --- | --- | --- | --- |
| B1 | 5554.69 | 1.86x10^^6^ | 1.13x10^^6^ (60.81) | 99.95 | 317 (174-505) |
| B9 | 153.52 | 1.48x10^^6^ | 0.17x10^^6^ (11.32) | 98.85 | 45 (17-90) |
| B13 | 542.97 | 1.94x10^^6^ | 0.84x10^^6^ (43.39) | 99.94 | 233 (121-390) |
| S35 | 5.75 | 1.56x10^^6^ | 0.00x10^^6^ (0.00) | 0.00 | 0 (0-0) |
| S45 | 6953.13 | 2.26x10^^6^ | 1.80x10^^6^ (78.74) | 99.95 | 504 (283-771) |
| S59 | 1019.53 | 2.78x10^^6^ | 1.90x10^^6^ (66.51) | 99.91 | 520 (287-819) |
| TF16 | 45.31 | 2.49x10^^6^ | 0.70x10^^6^ 26.12) | 99.92 | 173 (76-329) |
| TF34 | 44.92 | 1.93x10^^6^ | 0.30x10^^6^ (13.79) | 99.78 | 72 (31-136) |
| TF54 | 535.16 | 2.00x10^^6^ | 1.10x10^^6^ (53.30) | 99.95 | 299 (164-473) |
| G10 | 425.78 | 1.44x10^^6^ | 0.04x10^^6^  (2.46) | 27.80 | 4 (0-23) |
| G19 | 1789.06 | 1.80x10^^6^ | 0.07x10^^6^ (3.90) | 0.20 | 0 (0-2) |
| G24 | 197.27 | 1.97x10^^6^ | 0.10x10^^6^ (5.05) | 18.52 | 5 (0-14) |
| T8 | 39.84 | 1.51x10^^6^ | 0.03x10^^6^ (2.08) | 29.74 | 6 (0-22) |
| T64 | 58.20 | 1.89x10^^6^ | 0.07x10^^6^ (3.84) | 0.11 | 0 (0-0) |
| T94 | 2652.34 | 1.62x10^^6^ | 1.20x10^^6^ (72.36) | 99.95 | 331 (184-509) |
| J47 | 992.19 | 1.78x10^^6^ | 0.06x10^^6^ (3.61) | 0.89 | 0 (0-4) |
| J52 | 186.33 | 1.72x10^^6^ | 0.50x10^^6^ (27.92) | 99.93 | 132 (62-234) |
| A8 | 3906.25 | 2.36x10^^6^ | 1.50x10^^6^ (65.57) | 99.95 | 436 (247-674) |
| A9 | 44.92 | 1.58x10^^6^ | 0.01x10^^6^ (0.59) | 1.51 | 0 (0-5) |
| A15 | 742.19 | 2.13x10^^6^ | 1.30x10^^6^ (59.94) | 99.95 | 353 (189-580) |

**Genome copies, as determined by ddPCR*

***Ocular reference genome, A/HAR-13*

****Genome coverage at sites with depth >=10*
