# Supplementary figures and images for: Whole-genome sequencing of ocular Chlamydia trachomatis isolates from Gadarif State, Sudan
Source: Parasit Vectors. 2019 Nov 4;12:518. doi: 10.1186/s13071-019-3770-7 (PMC6829945; doi:10.1186/s13071-019-3770-7)

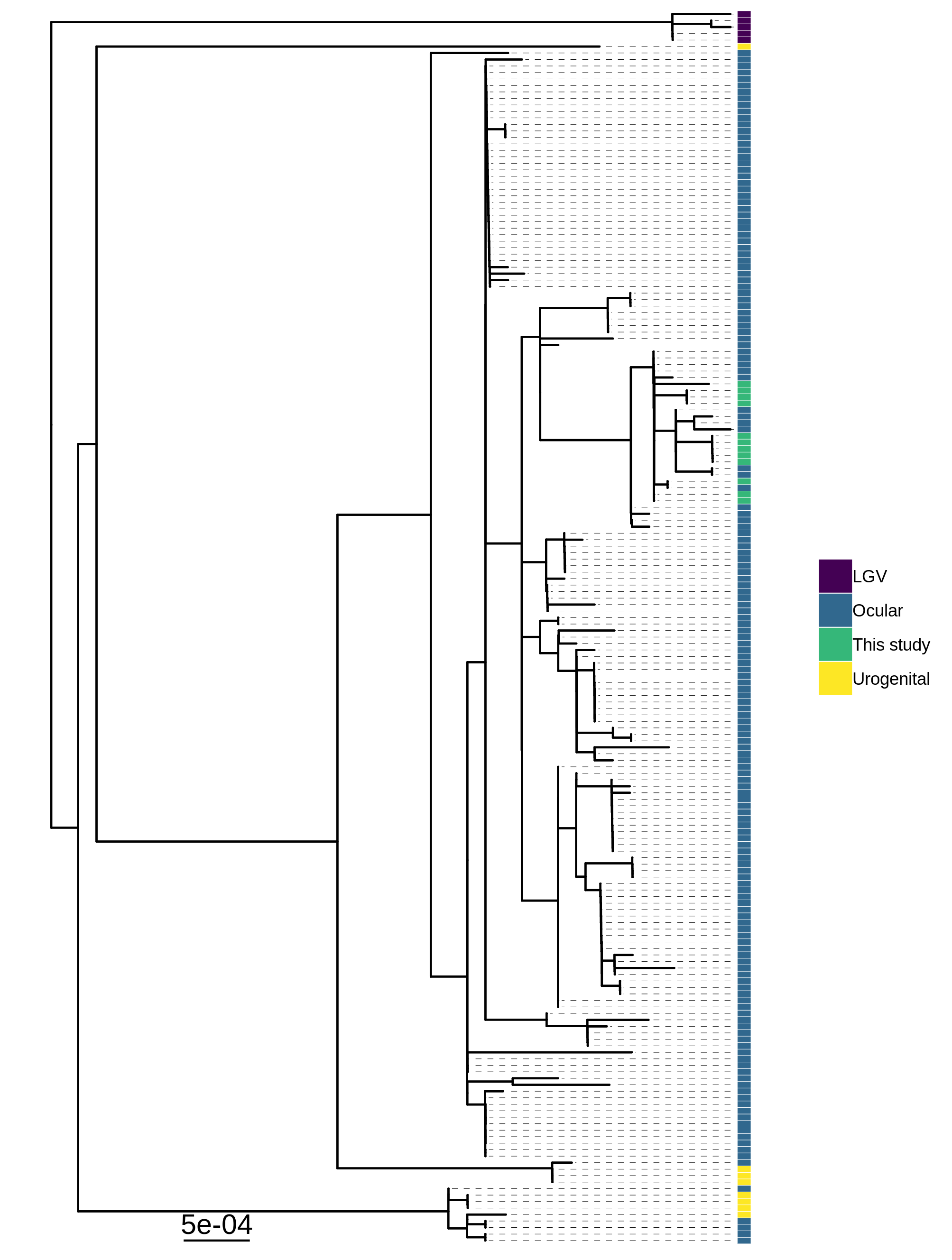

Supplement: Supplementary file 2 — Additional file 2: Figure S1. Maximum likelihood reconstruction of plasmid phylogeny of ocular Chlamydia trachomatis sequences from Sudan. Plasmid phylogeny of 12 C. trachomatis sequences from Sudan and 188 Ct clinical and reference strains. Sudanese C. trachomatis sequences were mapped to C. trachomatis A/HAR-13 using Bowtie2. SNPs were called using SAMtools/BCFtools. Phylogenies were computed with RAxML from a variable sites alignment using a GTR + gamma model and are midpoint rooted. The scale-bar indicates evolutionary distance. Sudanese C. trachomatis sequences in this study are coloured green, and reference strains are coloured by tissue localization (blue, ocular; yellow, urogenital; purple, LGV). [file 13071_2019_3770_MOESM2_ESM.tiff]
